# Equity in health insurance schemes enrollment in low and middle-income countries: A systematic review and meta-analysis

**DOI:** 10.1186/s12939-021-01608-x

**Published:** 2022-02-12

**Authors:** Doris Osei Afriyie, Blerina Krasniq, Brady Hooley, Fabrizio Tediosi, Günther Fink

**Affiliations:** 1 Department of Epidemiology and Public Health, Swiss Tropical and Public Health Institute, Kreuzstrasse 2, 4123 Allschwil, Switzerland; 2 University of Basel, Basel, Switzerland

**Keywords:** Health insurance, Low Income Population, Indigents Developing countries

## Abstract

**Background:**

Ensuring access to essential quality health services and reducing financial hardship for all individuals regardless of their ability to pay are the main goals of universal health coverage. Various health insurance schemes have been recently implemented in low- and middle-income countries (LMICs) to achieve both of these objectives. We systematically reviewed all available literature to assess the extent to which current health insurance schemes truly reach the poor and underserved populations in LMICs.

**Methods:**

In the systematic review, we searched on PubMed, Web of Science, EconLit and Google Scholar to identify eligible studies which captured health insurance enrollment information in LMICs from 2010 up to September 2019. Two authors independently selected studies, extracted data, and appraised included studies. The primary outcome of interest was health insurance enrollment of the most vulnerable populations relative to enrollment of the best-off subgroups. We classified households both with respect to their highest educational attainment and their relative wealth and used random-effects meta-analysis to estimate average enrollment gaps.

**Results:**

48 studies from 17 countries met the inclusion criteria. The average enrollment rate into health insurance schemes for vulnerable populations was 36% with an inter-quartile range of 26%. On average, across countries, households from the wealthiest subgroup had 61% higher odds (95% CI: 1.49 to 1.73) of insurance enrollment than households in the poorest group in the same country. Similarly, the most educated groups had 64% (95% CI: 1.32 to 1.95) higher odds of enrollment than the least educated groups.

**Conclusion:**

The results of this study show that despite major efforts by governments, health insurance schemes in low-and middle-income countries are generally not reaching the targeted underserved populations and predominantly supporting better-off population groups. Current health insurance designs should be carefully scrutinized, and the extent to which health insurance can be used to support the most vulnerable populations carefully re-assessed by countries, which are aiming to use health insurance schemes as means to reach their UHC goals. Furthermore, studies exploring best practices to include vulnerable groups in health insurance schemes are needed.

**Registration:**

Not available

**Supplementary Information:**

The online version contains supplementary material available at 10.1186/s12939-021-01608-x.

## Introduction

Improving equity in service utilization and ensuring financial protection for all individuals regardless of their ability to pay are key objectives within the global Universal health coverage (UHC) goals. Universal health coverage is of critical societal importance both in high- and in low-income settings, where inequalities between the rich and poor seem particularly large [1, 2]. Health insurance schemes are currently receiving increased attention globally not only as a health financing mechanism but also as a strategy to achieve universal health coverage [3] and as a means to reduce inequities between population groups [4].

In the absence of clear international guidelines, many low-and middle-income countries (LMICs) have started implementing a mix of social, national and community-based/mutual health insurance schemes over the past 15 years. Traditional social health insurance, which originated in Europe, uses earmarked payroll taxes from the formal sector as part of its health financing arrangements. Despite the generally small size of the formal sector, this type of health financing scheme has been adapted in many low-income settings, particularly in sub-Saharan Africa [5]. For example, in 2018, Zambia, which has an informal sector of almost 90%, passed the National Health Insurance bill which uses payroll taxes to improve access to quality health care for all its citizens [6, 7]. To extend health insurance coverage for those self-employed and the informal sector, community-based health insurance (CBHI) or mutual health insurance (MHI) have also emerged at various scales in Rwanda, Nepal, India, Burkina Faso, Cameroon, Mali and Senegal. CBHIs and MHI are typically voluntary schemes which target the informal sector and self-employed and their funds are pooled at the community level. Some countries such as Vietnam, Mexico and Peru have established noncontributory schemes using general tax revenues aimed at those not covered by social security schemes [8]. For example, in Thailand, there are three main health financing arrangements - a social security scheme for private formal sectors, a civil servants’ medical benefit scheme for civil servants and their families and a UHC scheme for those not affiliated with the other two schemes [9].

Current evidence of the impact of health insurance schemes in LMICs suggests some positive effects of insurance rollout on UHC goals [10–14]. Two recent reviews suggest that the reduction of financial barriers through CBHI and social health insurance improve service utilization and can protect its members from out-of-pocket expenditure [14, 15]. While these average impacts of health insurance schemes are certain, the extent to which these programs succeed in improving health and wellbeing of the most underserved population groups remains unclear [16, 17]. Knowledge and awareness of insurance programs, distance to health facilities, and payments associated with insurance schemes have been shown to be critical predictors of health insurance enrollment [18]. Meanwhile, these predicators might also undermine access of the most underserved groups to health insurance schemes.

In this manuscript, we systematically reviewed the literature on health insurance enrollment in LMICs to assess the extent to which current health insurance schemes reach poor and underserved populations.

## Methods

### Study Design

This study was designed as a systematic review and a meta-analysis of studies assessing the extent to which the most vulnerable populations are currently covered by health insurance schemes. We define vulnerable populations as the lowest group within the socioeconomic context (i.e., income, wealth quintile and education status in a country).

### Eligibility criteria

This review included randomized controlled trials, quasi-experimental, and observational studies related to health insurance enrollment in LMICs. The classification of countries as LMICs was based on the World Bank classification of per capita gross national income in 2019 (GNI per capita of $1,026 or less for low-income countries, GNI per capita between $1,026 and $3,995 for lower-middle income countries and for upper-middle income countries, the GNI per capita was $3,996-$12,376) [19]. We focused on studies that allowed the comparison of health insurance enrollment across groups with different socioeconomic status (income, wealth quintile, education status). We included health insurance schemes funded by the government including noncontributory health insurance and social health insurance schemes. Due to the popularity of community-based health insurances and mutual health insurances in LMICs, studies on such programs were included independent of their implementation scale. We restricted the studies to those published in English.

Studies were excluded if they only graphically displayed group differences in insurance enrollment. We also excluded papers exclusively focusing on private health insurance from the review. Studies which did not allow us to determine the type of health insurance (national, community-based, or private insurance schemes) were excluded.

### Search strategy

We conducted electronic searches from June 2019 to October 2019 in PubMed, Web of Science, EconLit (for economics literature) and Google Scholar. The search strategy relied on keywords from a combination of medical subject headings and free text including terms such as “health insurance”, “socioeconomic status”, “enroll” and “reach”. We filtered the search to studies published between January 2010 and September 2019 and were conducted in LMICs. We focused on studies published from 2010 since other systematic reviews on health insurance focused on earlier years [11, 14]. The search strategy for PubMed is shown in Table S1.

### Study selection and data extraction

Two independent authors screened all titles and abstracts of the initially identified articles to determine their eligibility for the inclusion criteria. The last author assisted in resolving any disagreement through a third review and after discussion with the review team. In the next phase, full articles were independently assessed for eligibility.

Two authors also independently extracted study information including type of scheme and its details, study design, year of data collection, relative enrollment rates of the poorest and least educated populations, type of point estimate and point estimate of enrollment for highest wealth and education groups compared to the lowest groups. Data were also extracted for non-overlapping populations (e.g. female vs male, urban vs rural). For studies that reported more than one adjusted point estimate, results from the least adjusted model were extracted in order to measure absolute enrollment gaps as consistently as possible.

### Quality assessment

In order to assess study quality and risk of bias, we adapted the National Institutes of Health (NIH) quality assessment tool for cross-sectional and case studies [20]. The tool contains fourteen parameters addressing internal and external validity concerns such as sample size justification, adjustment of potential confounding variables and participation rate of eligible persons. Given that we were primarily interested in absolute enrollment rates by population group rather than adjusted models, we removed items on the checklist related to confounding and analytical biases and added two questions on representativeness of the data set used, which we deemed to be of critical importance for our analysis.

### Data analysis

There were two stages in the analysis. First, we computed average enrollment rates of the poorest subpopulation as well as the absolute gaps in enrollment rates between the best-and worst-off subpopulations. In the second stage, we used random effects meta-analysis to analyze the odds of  health insurance enrollment of the group with lowest socioeconomic status relative to the subgroup with the highest socioeconomic status.

Given that multiple enrollment estimates were available for some countries, we first used random-effects meta-analysis to aggregate individual study estimates into a single pooled country estimate, and then conducted country-level meta-analysis using either the pooled estimate from the first step, or, for countries where only one study was available, the single country estimate. We assessed heterogeneity for adjustment in point estimates through subgroup analysis of those studies which had adjusted vs non adjusted odds ratio. We conducted all meta-analyses using STATA version 16 and illustrated results using forest plots.

## Results

### Search for studies

**Fig. 1:**
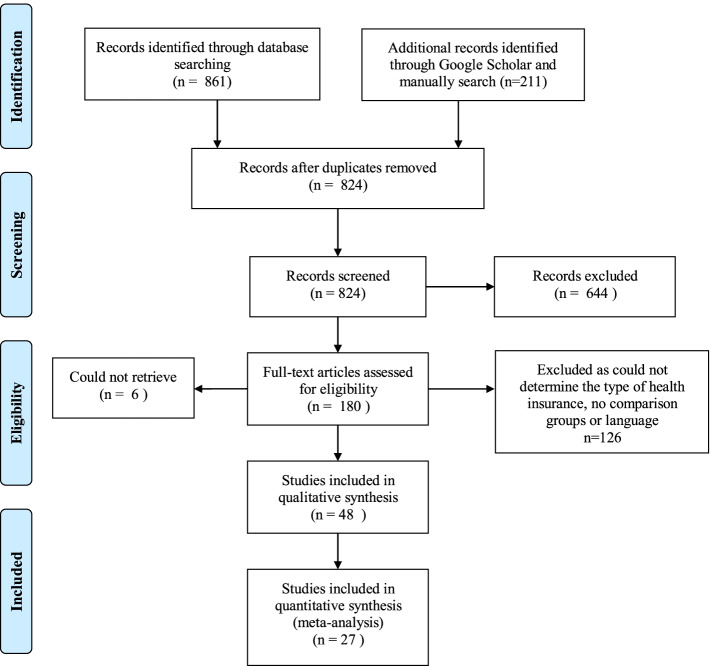
Flowchart of literature search

Figure 1 summarizes the main search process and results. Electronic searches of the four databases identified 1072 studies. After removing duplicates, 824 studies remained. 644 studies were excluded based on abstract and title review. There were 180 full text articles assessed for eligibility. Six studies were identified to be eligible for full-text assessment but they could not be retrieved. Hundred twenty-six studies did not report key variables of interest resulting in a final set of 48 studies.

### Characteristics of included studies

Almost all the studies (46/48) analyzed were single-country analyses (Table S2). Thirty-four of the single-country studies were from Sub-Saharan Africa. Notably, 23 studies were from Ghana, two studies each from Rwanda, South Africa, Burkina Faso and Tanzania, one study each in Kenya, Cameroon and Senegal. Twelve studies were conducted in Asia: three studies were from India, two studies each in Vietnam and Bangladesh and one study in Nepal, Laos, China, Sri Lanka, and Iran. There was only one study from South America (Colombia). One study analyzed both Ghana and Senegal [21]**.** Most of the studies (39/48) were published on from 2014-2018.

More than half of the studies (31/48) used primary data while the rest used representative household survey data. With regards to the primary outcome of interest, 39 studies examined health insurance enrollment by various education groups and 44 studies by wealth groups. For education, most studies (31/39) had four education categories: no formal education, primary education, secondary education, or higher education. For these groupings, enrollment rates were compared between those without any formal education and then those with a secondary or higher education. For income or wealth, majority of the studies (36/44) grouped households into quintiles and then enrollment rates were compared between the richest and poorest subgroups.

### Types of schemes and their policies for vulnerable groups

The included papers focused on 29 health insurance schemes in 17 countries as shown in Table 1. Most of the schemes (20/29) were implemented by either the central or sub-national government. The remaining 10 schemes were mutual or community health funds in which eight were organized by not-for-profit organizations and the other two by a research organization, and a health cooperative. Of the 25 schemes in which their year of establishment was reported by the studies, 21 were launched before the year 2010.

**Table 1:** Characteristics of schemes

Name of Scheme	Insurance Type	Country	Year	Entity responsible for scheme	Targeted Groups	Policy for Vulnerable groups
Amader Shasthya [22]	MHI	Bangladesh	2012	Research Organization	Chakaria sub district residents	Subsidy rate for the bottom 20% of the population
Labor Association for Social Protection [23]	MHI	Bangladesh	NR	Cooperative	Informal sector	NR
Assurance Maladie à Base Communautaire [24, 25]	CBHI	Burkina Faso	2004	District Government	Nouna District residents	Reduced premium for the poorest
Bamenda Ecclesiastical Provincial Health Assistance [26]	CBHI	Cameroon	NR	NPO	Residents of Bui and Donga-Mantung administrative divisions of North-West Cameroon	NR
New Rural Cooperative Medical Scheme [27]	SHI	China	2003	Central Government	Rural population	NR
Urban Employee Basic Medical Insurance [27]	SHI	China	1990	Central Government	Urban Employees	NR
Urban Resident Basic Medical Insurance [27]	SHI	China	2007	Central Government	Urban non-employees including adolescents and children	NR
Contributory social health insurance [28]	SHI	Colombia	NR	Central Government	Formal sector and their dependents	None
Subsidized Regime [28]	NCS*	Colombia	NR	Central Government	Low-income populations	Subsidies for lower-income populations
Ghana National Health Insurance [29–48]	SHI	Ghana	2003	Central Government	Whole Population	Exemptions for indigents, elderly, children
Jeevan Sanjivani [49]	MHI	India	2011	NPO	Kanpur Dehat Residents-rural area (among the poorest states in India)	NR
Rashtriya Swasthya Bima Yojana [50]	SHI	India	2008	State Government	Households below poverty level	NR
Sanjivan i[49]	MHI	India	2011	NPO	Pratapgarh Residents- rural area (among the poorest states in India)	NR
Swastha Kama l[49]	MHI	India	2011	NPO	Vaishali Residents- rural area (among the poorest states in India)	NR
Iran Health Insurance Organization [51]	SHI	Iran	NR	Central Government	Formal civil servants, informal and self-employed, residents of rural areas and small towns and minorities	Compulsory enrollment for groups that receive government subsidies
National Hospital Insurance Fund [52]	SHI	Kenya	1967	Central Government	Whole Population	100% subsidy through the Health Insurance Subsidy Program for the Poor
Community Health Fund [53]	CBHI	Laos	2001	Central Government	Self-employed & Informal sector	NR
Chandranigahapur Hospital of Rautahat district CBHI scheme [54]	CBHI	Nepal	2005/06	Central Government	Catchment area of Chandranigahapur Hospital	Subsidy rate
Mutuelle de santé [13, 55]	CBHI	Rwanda	1999	Central Government	Informal sector and rural population	Poorest 16% exempted from premium payment
Ndondol [56]	CBHI	Senegal	2001	NPO	Informal and agricultural population	NR
Plan Sesame [48]	NCS	Senegal	2006	Central Government	Older Population	NR
Soppante [56]	CBHI	Senegal	1997	NPO	Informal sector	NR
Wer Ak Werle [56]	CBHI	Senegal	2000	NPO	Informal traders	NR
Government Employees Medical Scheme [57, 58]	SHI	South Africa	2005	Central Government	Civil servants	Subsidy for low-income members
Multiple Micro-Insurance Companies [59]	MHI	Sri Lanka	NR	NPO	Poor households	NR
Community Health Fund [60]	CBHI	Tanzania	2001	District Government	Rural Informal Sector	Exemptions for poor households
Compulsory Health Insurance [61]	SHI	Vietnam	1993	Central Government	Civil servants, formal sector, pensioners, children below six years	NR
Health Care for the poor [61]	NCS	Vietnam	2003	Central Government	Poor & ethnic minorities	100% subsidy for the poor
Student Health Insurance [62]	SHI	Vietnam	1995	Central Government	Students	None

*NCS-Non-contributory scheme, NPO-Not-for-profit Organization, NR-Not reported, SHI-Social Health Insurance

A majority of the schemes (25/29) were designed to target specifically the informal sector, poor households, and rural populations and/or provide either premium subsidization or exemption to vulnerable populations. Among these 25 schemes, three (Health Care for the poor in Vietnam, Subsidized Health Insurance in Colombia, and the Kenya National Hospital Insurance Fund) were established to provide 100% subsidy to the poor. The other five schemes were implemented by the central government which are targeted for specific groups such as the formal sector, students, and urban residents.

### Health insurance enrollment rate among the most vulnerable groups

**Fig. 2:**
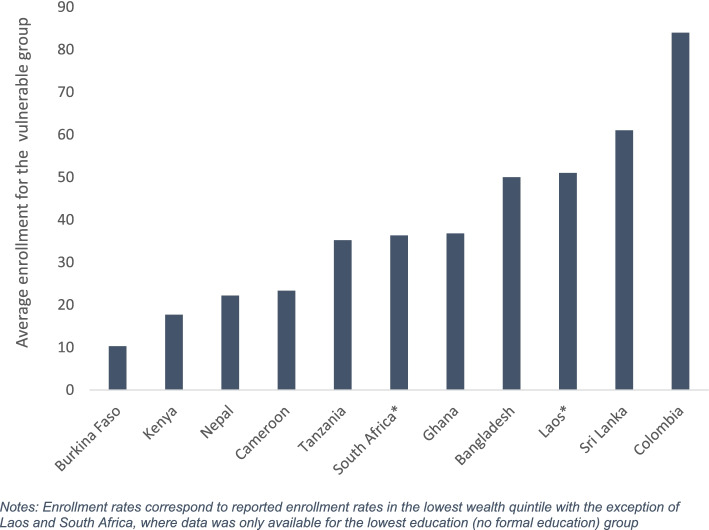
Average health insurance enrollment rate for the vulnerable population by country

The enrollment rate into any type of health insurance scheme among the most vulnerable population group was 36% on average with an inter-quartile range of 28%. The enrollment rate varied from 10.3% in a district mutual fund in Burkina Faso to 87.8% in the subsidized regime of Colombia’ social health insurance which targets the poor (Figure 2). Furthermore, households in the lowest wealth or income quintile were on average 19 percentage points less likely to enroll compared to households in the highest socioeconomic group (Figure S1).

**Fig. 3:**
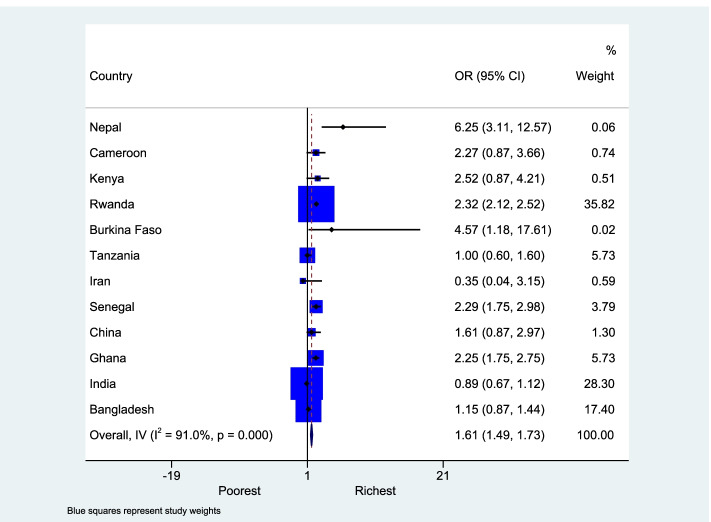
Forest plot showing the meta-analysis of all countries for health insurance enrollment between the highest and lowest wealth groups

After data extraction, a total of 31 studies from 13 countries reporting odds ratio or logit coefficient comparing highest and lowest groups for wealth and education in health insurance enrollment were included in the meta-analysis. For wealth status, point estimates of the relative insurance enrollment were available from 28 studies covering 12 countries (Figure 3). Multiple point estimates were available for Bangladesh, Cameroon, Ghana, India, and Kenya. Figure S2 shows the results of the random effect meta-analysis used to create a single country-specific estimate for these countries. Across countries, households from the wealthiest subgroup had on average 61% higher odds (95% CI: 1.49 to 1.73) of enrollment into health insurance schemes than households in the poorest group of the same country.

**Fig. 4:**
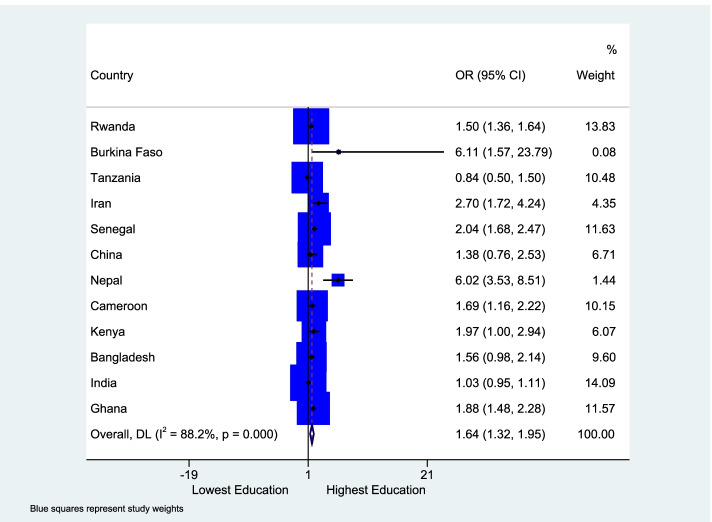
Forest plot showing the meta-analysis of all countries for health insurance enrollment between the highest and lowest educated groups

There was high heterogeneity across countries (I-squared=91.0%; p-value<0.01). Most (8) of the countries had an odds ratio of enrollment for the richest groups to be over two times the odds of the enrollment for the poorest groups. Only the health insurance schemes in Iran and India had an odds ratio less than one (OR: 0.35, 95% CI: 0.04 to 3.15 and OR: 0.89, 95% CI: 0.67 to 1.12, respectively). The same patterns emerged when we examined enrollment status by educational attainment group. The enrollment gap between the least and most educated groups ranged from -6.9% to -41.2% (Figure S3), with an average gap of about 19 % percentage points. Point estimates of the relative insurance enrollment for education groups were available for 25 studies in 12 countries. As shown in Figure 4, the most educated groups had on average 64% (95% CI: 1.32 to 1.95) higher odds of enrollment than the least educated groups. The CBHI scheme in Burkina Faso had the highest odds ratio of 6.11 for the enrollment for the most educated compared to the least educated, whilst the lowest odds ratio between these two groups was 0.84 in Tanzania. There was high heterogeneity between studies (I-squared=88.2%; p-value<0.01). There were six countries, Bangladesh, Cameroon, Ghana, India, Kenya and Nepal, which had estimates from multiple studies for education groups (Figure S4).

Subgroup analysis for education comparing studies with crude versus studies with adjusted ORs showed some differences. The pooled unadjusted odds ratio was 2.32 (95% CI: 1.42 to 3.23) compared to the pooled odds ratio of 1.44 (95% CI: 1.24 to 1.63) in studies adjusting for sex, age, ethnicity, location, marital status, household size, religion, health status and employment status (Figure S5).

### Quality assessment

All the studies included in the review were observational. Of the 48 studies, 20 were rated as ‘good’, whilst 27 were rated ‘fair’ (Table 2). Only one study was rated as ‘poor’. This study was removed from analysis. The alternative estimates with the full sample are included in File S6. All the studies had the basic elements related to having a clear research question, a defined study population, and selection criteria of participants. However, only 10 studies reported the participation rate of eligible persons. Few studies relied on large administrative population-based data. Studies were rated as ‘fair’ if the study population was not representative of the general population.

**Table 2:** Study quality

Criteria		Yes
1	Was the research question or objective in this paper clearly stated?	48/48
2	Was the study population clearly specified and defined?	48/48
3	Was the participation rate of eligible persons at least 50%?	17/48
4	Were all the subjects selected or recruited from the same or similar populations (including the same time period)? Were inclusion and exclusion criteria for being in the study pre-specified and applied uniformly to all participants	48/48
5	Was the study population similar to the national population?	14/48
6	Was the sampling methods specified and appropriate?	48/48

## Discussion

We conducted a systematic review with the aim of assessing the extent to which health insurance schemes are currently reaching the most vulnerable population groups in LMICs. We found 48 studies, which focused on 29 health insurance schemes from 17 LMICs allowing us to compare enrollment across socioeconomic groups, most of which were published after 2013. Overall, the results of our review are clear: current health insurance schemes reach only a relatively small proportion of the most vulnerable population groups.

The only scheme in which the enrolment rate was far lower for the wealthiest populations was the Colombian subsidized regime which exclusively targeted the poor and other vulnerable groups as those in the formal sector and self-employed workers with a steady income are required to obtain the contributory regime. Two features of the scheme which seem important are, first, that the scheme is mandatory for all those who are eligible to enroll. Second, during the period of analysis by Ruiz-Gomez et al, municipalities were using a mean proxy test to select beneficiaries into the scheme through the established social service beneficiaries’ identification system (Sistema de Identificación de los Beneficiarios de los Servicios Sociales, SISBEN )[63].

Even though virtually all the other insurance schemes analyzed directly target or subsidize the most vulnerable groups, better-off households have on average almost twice the odds of enrolling in health insurance compared to poorest households. For example, the Ghana health insurance scheme stipulates premium exemptions for indigents, the elderly above 70, pregnant women and children while the Rwanda Mutelle de Santé exempts the poorest 16% of households from premium payments. Other schemes such as those implemented in Nepal, Bangladesh and Burkina Faso have subsidized rates for the poorest households.

Despite these efforts, enrollment rates of the wealthiest subpopulations are higher than those of the most vulnerable population groups in all of these countries. These results are consistent with previous work on health insurance programs showing that enrollment and willingness to purchase health insurance in LMICs is pro-rich, which are explained by factors such as greater exposure of the rich to the media and their higher income levels to pay for health insurance premiums [14, 51, 64].

The current enrollment gaps should not necessarily be interpreted as evidence that current targeting efforts do not make enrollment easier for poor households. Rather, it demonstrates that these current measures appear insufficient to equitably include vulnerable populations in health insurance schemes. Given that most health insurance schemes in LMICs are heavily financed by central government revenues, the currently observed enrollment patterns essentially make health insurance a regressive policy, primarily subsidizing health care for better-off households. Further reductions in premiums and improving geographical access to health facilities could potentially increase uptake among poor and underserved populations [12]; other policy options include automatic (free) insurance enrollment of these groups or the direct provision of free health services for these groups.

Despite our best effort to review all of the recent evidence available, the findings presented in this manuscript have limitations. First, the included studies were retrospective and cross-sectional, and primarily focused on CBHI and national health insurance schemes. Second, due to the language restriction for publications in English, there was a limitation by the exclusion of articles published in other languages. In the past two decades, many LMICs in Latin America have implemented health insurance schemes such as non-contributory schemes for vulnerable populations [65]. Therefore, restricting the literature search to English may have underrepresented the inclusion of studies from this region which in turn may have underestimated health insurance enrollment of vulnerable populations. Thirdly, it was also quite striking that nearly half (23/48) of all studies identified focused on Ghana, while no studies were found on several other countries where similar insurance programs have been launched in the recent past. In addition, studies used highly heterogeneous ways of measuring wealth or income that may not be directly comparable. Our analysis also pooled data across different designs of insurance schemes and socioeconomic group definitions and therefore, represents an average across highly heterogeneous systems. In addition, nearly all the studies relied on self-reported data about wealth or income and educational status which could lead to misclassification due to recall bias. Lastly, another limitation of our study is the lack of longitudinal data that would have allowed evaluating whether there are countries that are successfully reducing inequalities in health insurance enrollment. Large longitudinal trend studies are needed to determine the contribution of health insurance schemes in reducing inequalities between the rich and poor over time

Despite these limitations, our findings are consistent with a larger analysis of the World Health Surveys conducted in the early 2000s which suggested that health insurance schemes continue to primarily benefit the better-off populations [66]. In their current form, health insurance schemes are thus unlikely to be viable mechanisms to promote universal health coverage. Challenges faced by current schemes include difficulties associated with identifying the most poor or vulnerable populations [67–70] as well as management of rollout and implementation at sub-national levels [71]. Increased financial, political, and institutional resources are likely needed to identify and reach underserved populations. In addition, simplified administrative processes for enrollment such as automatic enrollment after their identification could also facilitate the inclusion of underserved populations [72–74].

## Conclusion

Although all recently introduced health insurance schemes LMICs aim at providing access to health services as well as financial protection to the most vulnerable populations, current coverage is low among the poor, and highly regressive in most countries. Experiences from countries suggest that current strategies to improve coverage of vulnerable populations in health insurance schemes have not achieved their aim of equity. Further investigation is needed to understand why these strategies are not reaching vulnerable groups. The evidence also suggests countries that are planning to establish health insurance schemes with the aim of equity for vulnerable populations might need to reevaluate their approach given the findings of this review.

## Supplementary Information

**Additional file 1. Table S1. **PubMed search strategy.

**Additional file 2. Table S2. **Characteristics of included studies.

**Additional file 3. Figure S1. **Absolute percentage enrollment gap at the population level between the lowest and highest wealth groups.

**Additional file 4. Figure S2. **Forest plot showing countries with multiple estimates.

**Additional file 5. Figure S3. **Absolute percentage enrollment gap at the population level between the least and most educated groups.

**Additional file 6. Figure S4. **Forest plot showing countries with multiple estimates for education.

**Additional file 7. Figure S5. **Forest plot showing subgroup analysis of adjusted and crude odds ratio.

**Additional file 8. File S6.** Figures showing estimates regardless of quality rating.

## Data Availability

The data analyzed is available from the corresponding author on reasonable request.

## Abbreviations

CBHI: Community based health insurance
LMICs: Low-and Middle-income countries
MHI: Mutual Health Insurance
NCS: Non-Contributory Scheme
NPO: Not-for-profit
NR: Not Reported
SHI: Social Health Insurance
UHC: Universal Health Coverage
