# Supplementary material for: Equity in health insurance schemes enrollment in low and middle-income countries: A systematic review and meta-analysis
Source: Int J Equity Health. 2022 Feb 12;21:21. doi: 10.1186/s12939-021-01608-x (PMC8841076; doi:10.1186/s12939-021-01608-x)
Supplement: Supplementary file 2 — Additional file 2. Table S2. Characteristics of included studies. [file 12939_2021_1608_MOESM2_ESM.docx]

**Table S2: Characteristics of included studies**

| **Author_Year** | **Country** | **Data Type** | **Study design** | **Sample size** | **Enrollment for highest vs lowest education group** | **Enrollment for best vs worst off group** |
| --- | --- | --- | --- | --- | --- | --- |
| Adhikari_2013[1] | Nepal | Primary | Case Control | 416 | Educated vs Uneducated | Quintile 5 vs Quintile 1 |
| Akazili_2014[2] | Ghana | Primary | Cross-sectional | 5469 | Secondary/Higher vs None | Quintile 5 vs Quintile 1 |
| Fenny_2017[3] | Ghana | Primary | Cross-sectional | 758 | Secondary/Higher vs None | Quintile 5 vs Quintile 1 |
| Mahmood_2018[4] | Bangladesh | Primary | Case Control | 1956 | 10+ years vs None | Quintile 5 vs Quintile 1 |
| Macha_2014[33] | Tanzania | Primary | Cross-sectional | 1225 | Secondary/Higher vs None | Quintile 5 vs Quintile 1 |
| Alkenbrack_2013[5] | Laos | Primary | Case-Control | 3000 | University vs Any primary | Quintile 5 vs Quintile 1 |
| Amo_2014[6] | Ghana | Primary | Cross-sectional | 210 | University vs None | Quintile 5 vs Quintile 1 |
| Kotoh_2016[7] | Ghana | Primary | Cross-sectional | 6790 | None | Quintile 5 vs Quintile 1 |
| Dixon_2014[8] | Ghana | Primary | Cross-sectional | 2119 | Secondary/Higher vs None | Quintile 5 vs Quintile 1 |
| Jehu-Appiah_2011[9] | Ghana | Primary | Cross-sectional | 3301 | None | Quintile 5 vs Quintile 1 |
| Duku_2015[10] | Ghana | Primary | Cross-sectional | 4214 | None | Quintile 5 vs Quintile 1 |
| Nguyen_2013[11] | Vietnam | Vietnam Household Living Standard Survey | Cross-sectional | 3526 | University vs None | Quintile 5 vs Quintile 1 |
| Parmar_2014[12] | Burkina Faso | Household Survey | Cross-sectional | 4695 | Literate vs Illiterate | Quart 2-4 vs Quart 1 |
| Kusi_2018[13] | Ghana | Primary | Cross-sectional | 3173 | Secondary/Higher vs None | Quintile 5 vs Quintile 1 |
| Panda_2014[14] | India | Primary | Cross-sectional | 433 | None | Quintile 5 vs Quintile 1 |
| Panda_2014[14] | India | Primary | Cross-sectional | 378 | None | Quintile 5 vs Quintile 1 |
| Panda_2014[14] | India | Primary | Cross-sectional | 524 | None | Quintile 5 vs Quintile 1 |
| Kusi_2015 [15] | Ghana | Primary | Cross-sectional | 2418 | None | Quintile 5 vs Quintile 1 |
| Kumi-Kyereme_2013 [16] | Ghana | Ghana DHS | Cross-sectional | 4910 | University vs None | Quintile 5 vs Quintile 1 |
| Duku_2018[17] | Ghana | Primary | Cross-sectional | 4214 | Post University vs Primary | Quintile 5 vs Quintile 1 |
| Amu_2016[18] | Ghana | Ghana DHS | Cross-sectional | 9263 | University vs None | Quintile 5 vs Quintile 1 |
| Dixon_2011[19] | Ghana | Ghana DHS | Cross-sectional | 9479 | Secondary/Higher vs None | Quintile 5 vs Quintile 1 |
| Bendig_2011[20] | Sri Lanka | Primary | Case Control | 330 | University vs None | Quintile 5 vs Quintile 1 |
| Sarpong_2010 [21] | Ghana | Primary | Cross-sectional | 7225 | None | Trisect 3 vs Trisect 1 |
| Seddoh_2018[22] | Ghana | Household Survey | Cross-sectional | 625 | Post-University vs None | None |
| Duku_2018[23] | Ghana | Primary | Cross-sectional | 4214 | Secondary/Higher vs None | Quintile 5 vs Quintile 1 |
| Oraro_2018[24] | Cameroon | Primary | Cross-sectional | 930 | Secondary/Higher vs Primary or less | Quintile 5 vs Quintile 1-2 |
| Van der Wielen_2018[25] | Ghana | Ghana Living Standard Survey | Cross-sectional | 4086 | Secondary/Higher vs None | Bisect 2 vs Bisect 1 |
| Oraro_2018[26] | Kenya | Primary | Cross-sectional | 444 | Secondary/Higher vs Primary or less | Quintile 5 vs Quintile 1-2 |
| Nguyen_2010[27] | Vietnam | National Health Survey | Cross-sectional | 27563 | University vs None | Quintile 5 vs Quintile 1 |
| Dror_2018[28] | India | Primary | Cross-sectional | 524 | None | Quintile 5 vs Quintile 3 |
| Kotoh_2018 [29] | Ghana | Household Survey | Cross-sectional | 6790 | None | Quintile 5 vs Quintile 1 |
| Finnoff_2010 [30] | Rwanda | Integrated Living Conditions Survey | Cross-sectional | 34785 | University vs None | Quintile 5 vs Quintile 1 |
| Khalid_2017[31] | Ghana | Socioeconomic Panel Survey | Cross-sectional | 5761 | University vs None | Quintile 5 vs Quintile 1 |
| Lu_2012 [32] | Rwanda | Integrated Living Conditions Survey | Cross-sectional | 13320 | Less than Primary vs None | Quintile 5 vs Quintile 1 |
| Govender_2013 [33] | South Africa | Primary | Cross-sectional | 1329 | University vs None/Primary | None |
| Boateng_2013 [34] | Ghana | Primary | Cross-sectional | 300 | University vs None | Quart 4 vs Quart 1 |
| Cofie_2013[35] | Burkina Faso | Household Survey | Cross-sectional | 250 | Secondary/Higher vs None | None vs Radio or TV |
| Manortey_2014 [36] | Ghana | Primary | Cross-sectional | 3228 | University vs None | Trisect 3 vs Trisect 1 |
| Mladovsky_2014[37] | Senegal | Primary | Case Control | 241 | Secondary/Higher vs None | Quintile 5 vs Quintile 1 |
| Jin_2016[38] | China | China Health and Longitudinal Survey | Cross-sectional | 18605 | Secondary/Higher vs None | Quintile 5 vs Quintile 1 |
| Kapologwe_2017 [39] | Tanzania | Primary | Cross-sectional | 460 | Secondary/Higher vs None/Primary | Bisect 2 vs Bisect 1 |
| Sarker_2017[40] | Bangladesh | Primary | Case Control | 784 | University vs None | Quintile 5 vs Quintile 1 |
| Goudge_2018 [41] | South Africa | Primary | Cross-sectional | 1329 | University vs None/Primary | None |
| Van der Wielen_2018 | Ghana | Ghana Living Standard Survey | Cross-sectional | 5846 | Secondary/Higher vs None | Quintile 5 vs Quintile 1 |
| Ruiz Gomez_2013 [42] | Colombia | Colombian Life Quality Survey | Cross-sectional | NA | None | Quintile 5 vs Quintile 1 |
| Ghosh_2014 [43] | India | Primary | Cross-sectional | 6000 | None | Quintile 5 vs Quintile 1 |
| Nosratnejad_2016 | Iran | National Health Survey | Cross-sectional | 23543 | University vs Primary or less | Quintile 5 vs Quintile 1 |
| Kuuire_2017][44] | Ghana | Global Ageing and Health Survey | Cross-sectional | 1534 | University vs None | Quintile 5 vs Quintile 1 |
| Parmar_2014[45] | Ghana | Primary | Cross-sectional | 435 | Educated vs Uneducated | Quart 4 vs Quart 1 |
| Parmar_2014[45] | Senegal | Primary | Cross-sectional | 2933 | Educated vs Uneducated | Quart 4 vs Quart 2 |

**References**

1. Adhikari N, Wagle RR, Adhikari DR, Thapa P, Adhikari M: **Factors Affecting Enrolment in the Community Based Health Insurance Scheme of Chandranigahapur Hospital of Rautahat District**. *Journal of Nepal Health Research Council* 2019, **16**(41):378-384.

2. Akazili J, Welaga P, Bawah A, Achana FS, Oduro A, Awoonor-Williams JK, Williams JE, Aikins M, Phillips JF: **Is Ghana's pro-poor health insurance scheme really for the poor? Evidence from Northern Ghana**. *BMC Health Services Research* 2014, **14**(637):1-9.

3. Fenny AP: **Live to 70 Years and Older or Suffer in Silence: Understanding Health Insurance Status Among the Elderly Under the NHIS in Ghana**. *Journal of aging & social policy* 2017, **29**(4):352-370.

4. Mahmood SS, Hanifi SMA, Mia MN, Chowdhury AH, Rahman M, Iqbal M, Bhuiya A: **Who enrols in voluntary micro health insurance schemes in low-resource settings? Experience from a rural area in Bangladesh**. *Global Health Action* 2018, **11**(1):1-10.

5. Alkenbrack S, Jacobs B, Lindelow M: **Achieving universal health coverage through voluntary insurance: what can we learn from the experience of Lao PDR?** *BMC health services research* 2013, **13**(1):521-521.

6. Amo T: **The National Health Insurance Scheme (NHIS) in the Dormaa Municipality, Ghana: why some residents remain uninsured?** *Global journal of health science* 2014, **6**(3):82-89.

7. Kotoh AMAM, … SVdGfei, Undefined, Van der Geest S: **Why are the poor less covered in Ghana's national health insurance? A critical analysis of policy and practice**. *equityhealthjbiomedcentralcom* 2016, **15**(1):34-46.

8. Dixon J, Luginaah I: **Determinants of Health Insurance Enrolment in Ghana' s Upper West Region**. In*.*; 2014: 259-259.

9. Jehu-Appiah C, Aryeetey G, Spaan E, de Hoop T, Agyepong I, Baltussen R: **Equity aspects of the National Health Insurance Scheme in Ghana: Who is enrolling, who is not and why?** *SOCIAL SCIENCE & MEDICINE* 2011, **72**(2):157-165.

10. Duku SKOSKO, van Dullemen CE, Fenenga C: **Does health insurance premium exemption policy for older people increase access to health care? Evidence from Ghana**. *JOURNAL OF AGING & SOCIAL POLICY* 2015, **27**(4):331-347.

11. Nguyen THT-H, Leung S: **Dynamics of Health Insurance Enrollment in Vietnam, 2004-2006**. *Journal of the Asia Pacific Economy* 2013, **18**(4):594-614.

12. Parmar D, De Allegri M, Savadogo G, Sauerborn R, Allegri MD: **Do community-based health insurance schemes fulfil the promise of equity? A study from Burkina Faso**. *HEALTH POLICY AND PLANNING* 2014, **29**(1):76-84.

13. Kusi A, Fenny A, Arhinful DK, Asante FA, Parmar D: **Determinants of enrolment in the NHIS for women in Ghana – a cross sectional study**. *International Journal of Social Economics* 2018, **45**(9):1318-1334.

14. Panda P, Chakraborty A, Dror DM, Bedi AS: **Enrollment in Community-Based Health Insurance Schemes in Rural Bihar and Uttar Pradesh, India**. *Health Policy and Planning* 2014, **29**(8):960-974.

15. Kusi A, Enemark U, S HK, A AF: **Refusal to enrol in Ghana's National Health Insurance Scheme: is affordability the problem?** *International Journal for Equity in Health* 2015, **14**(2):14-14.

16. Kumi-Kyereme A, Amo-Adjei J, Health JA-A: **Effects of spatial location and household wealth on health insurance subscription among women in Ghana**. *BMC Health Services Research* 2013, **13**(1):221-229.

17. Duku SKO, Fenenga CJ, Alhassan RK, Nketiah-Amponsah E: **Rural-urban differences in the determinants of enrolment in health insurance in Ghana**. *BMC HEALTH SERVICES RESEARCH* 2018, **18**(384):1-23.

18. Amu H, Dickson KS: **Health insurance subscription among women in reproductive age in Ghana: do socio-demographics matter?** *Health Economics Review* 2016, **6**(24):1-8.

19. Dixon J, … EYTE, Planning, Undefined: **Ghana's National Health Insurance Scheme: helping the poor or leaving them behind?** *journalssagepubcom* 2011.

20. Bendig M, Arun T: **Enrolment in Micro Life and Health Insurance: Evidences from Sri Lanka**. *Econstor* 2011(5427):1-30.

21. Sarpong N, Loag W, Fobil J, Meyer CG, Adu-Sarkodie Y, May J, Schwarz NG: **National health insurance coverage and socio-economic status in a rural district of Ghana**. *Tropical Medicine and International Health* 2010, **15**(2):191-197.

22. Seddoh A, Sataru F: **Mundane? Demographic characteristics as predictors of enrolment onto the National Health Insurance Scheme in two districts of Ghana**. *BMC Health Services Research* 2018, **18**(1):330-336.

23. Duku SKO: **Differences in the determinants of health insurance enrolment among working-age adults in two regions in Ghana**. *BMC HEALTH SERVICES RESEARCH* 2018, **18**(384):1-16.

24. Oraro T, Ngube N, Atohmbom GY, Srivastava S, Wyss K: **The influence of gender and household headship on voluntary health insurance: the case of North-West Cameroon**. *Health Policy and Planning* 2018, **33**(2):163-170.

25. van der Wielen N, Channon AA, Falkingham J: **Does insurance enrolment increase healthcare utilisation among rural-dwelling older adults? Evidence from the National Health Insurance Scheme in Ghana**. *BMJ GLOBAL HEALTH* 2018, **3**(1):1-9.

26. Oraro T, Wyss K: **How does membership in local savings groups influence the determinants of national health insurance demand? A cross-sectional study in Kisumu, Kenya**. *INTERNATIONAL JOURNAL FOR EQUITY IN HEALTH* 2018, **17**(1):170-170.

27. Nguyen H, Knowles J: **Demand for voluntary health insurance in developing countries: The case of Vietnam's school-age children and adolescent student health insurance program**. *SOCIAL SCIENCE & MEDICINE* 2010, **71**(12):2074-2082.

28. Dror DM, Majumdar A, Chakraborty A: **The effect of consensus on demand for voluntary micro health insurance in rural India**. *RISK MANAGEMENT AND HEALTHCARE POLICY* 2018, **11**:139-158.

29. Kotoh AM, Aryeetey GC, der Geest S, Van der Geest S: **Factors That Influence Enrolment and Retention in Ghana' National Health Insurance Scheme**. *INTERNATIONAL JOURNAL OF HEALTH POLICY AND MANAGEMENT* 2018, **7**(5):443-454.

30. Finnoff C: **Gendered Vulnerabilities after Genocide: Three Essays on Post-conflict Rwanda**. University of Massachusetts; 2010.

31. Khalid M: **Three Essays on the Informal Sector**. University of Manitoba; 2017.

32. Lu C, Chin B, Lewandowski JL, Basinga P, Hirschhorn LR, Hill K, Murray M, Binagwaho A: **Towards universal health coverage: an evaluation of Rwanda Mutuelles in its first eight years**. *PloS one* 2012, **7**(6):1-16.

33. Govender V, Chersich MF, Harris B, Alaba O, Ataguba JE, Nxumalo N, Goudge J: **Moving towards universal coverage in South Africa? Lessons from a voluntary government insurance scheme**. *Global Health Action* 2013, **6**(1):109-119.

34. Boateng D, Awunyor-Vitor D: **Health insurance in Ghana: evaluation of policy holders' perceptions and factors influencing policy renewal in the Volta region**. *International journal for equity in health* 2013, **12**(50):1-10.

35. Cofie P, De Allegri M, Kouyaté B, Sauerborn R: **Effects of information, education, and communication campaign on a community-based health insurance scheme in Burkina Faso**. *Global health action* 2013, **6**(1):1-12.

36. Manortey S, VanDerslice J, Alder S, Henry KA, Crookston B, Dickerson T, Benson S: **Spatial Analysis of Factors Associated with Household Subscription to the National Health Insurance Scheme in Rural Ghana**. *Journal of public health in Africa* 2014, **5**(1):353-361.

37. Mladovsky P, Soors W, Ndiaye P, Ndiaye A, Criel B: **Can social capital help explain enrolment (or lack thereof) in community-based health insurance? Results of an exploratory mixed methods study from Senegal**. *Social science & medicine (1982)* 2014, **101**:18-27.

38. Jin Y, Hou Z, Zhang D: **Determinants of Health Insurance Coverage among People Aged 45 and over in China: Who Buys Public, Private and Multiple Insurance**. *PLOS ONE* 2016, **11**(8):1-15.

39. Kapologwe NA, Kagaruki GB, Kalolo A, Ally M, Shao A, Meshack M, Stoermer M, Briet A, Wiedenmayer K, Hoffman A: **Barriers and facilitators to enrollment and re-enrollment into the community health funds/Tiba Kwa Kadi (CHF/TIKA) in Tanzania: a cross-sectional inquiry on the effects of socio-demographic factors and social marketing strategies**. *BMC Health Services Research* 2017, **17**(1):308-308.

40. Sarker AR, Sultana M, Mahumud RA, Ahmed S, Islam Z, Morton A, Khan JAM: **Determinants of enrollment of informal sector workers in cooperative based health scheme in Bangladesh**. *PLOS ONE* 2017, **12**(7):1-12.

41. Goudge J, Alaba OA, Govender V, Harris B, Nxumalo N, Chersich MF: **Social health insurance contributes to universal coverage in South Africa, but generates inequities:a survey among members of a government employee insurance scheme**. *International journal for Equity in Health* 2018, **17**(1):1-13.

42. Van der Wielen N, Channon AA, Falkingham J: **Universal health coverage in the context of population ageing: What determines health insurance enrolment in rural Ghana?** *BMC Public Health* 2018, **18**(1):657-670.

43. Ghosh S: **Publicly-Financed Health Insurance for the Poor Understanding RSBY in Maharashtra**. 2014, **46**.

44. Kuuire VZ, Tenkorang EY, Rishworth A, Luginaah I, Yawson AE: **Is the Pro-Poor Premium Exemption Policy of Ghana's NHIS Reducing Disparities Among the Elderly?** *POPULATION RESEARCH AND POLICY REVIEW* 2017, **36**(2):231-249.

45. Parmar D, Williams G, Dkhimi F, Ndiaye A, Asante FA, Arhinful DK, Mladovsky P: **Enrolment of older people in social health protection programs in West Africa--does social exclusion play a part?** *Social science & medicine (1982)* 2014, **119**:36-44.
