# Supplementary material for: Equity in health insurance schemes enrollment in low and middle-income countries: A systematic review and meta-analysis
Source: Int J Equity Health. 2022 Feb 12;21:21. doi: 10.1186/s12939-021-01608-x (PMC8841076; doi:10.1186/s12939-021-01608-x)
Supplement: Supplementary file 3 — Additional file 3. Figure S1. Absolute percentage enrollment gap at the population level between the lowest and highest wealth groups. [file 12939_2021_1608_MOESM3_ESM.docx]

**Figure S1. Absolute percentage enrollment gap at the population level between the lowest and highest wealth groups**

Of the 48 studies reviewed, 20 studies from eight countries collected data on absolute health insurance enrollment differences at the population level between the highest and lowest groups. However, after removing the one study, which was of low quality, 19 studies remained. Only two studies from Colombia and Ghana [1, 2] reported a higher percentage of enrollment for the lowest wealth groups than the highest wealth groups. Among these studies, the highest enrollment difference was 77.89% whilst the lowest was 1.7%. The 17 studies, which reported a lower enrollment for the lowest wealth groups, enrollment gaps ranged from -70.84% to -6.2%.

**References**

1. Ruiz Gomez F, Zapata Jaramillo T, Garavito Beltran L: **Colombian health care system: results on equity for five health dimensions, 2003-2008**. *Rev Panam Salud Publuca* 2013, **33**(2):107-199.

2. Boateng D, Awunyor-Vitor D: **Health insurance in Ghana: evaluation of policy holders' perceptions and factors influencing policy renewal in the Volta region**. *International journal for equity in health* 2013, **12**(50):1-10.
