# Supplementary figures and images for: Equity in health insurance schemes enrollment in low and middle-income countries: A systematic review and meta-analysis
Source: Int J Equity Health. 2022 Feb 12;21:21. doi: 10.1186/s12939-021-01608-x (PMC8841076; doi:10.1186/s12939-021-01608-x)

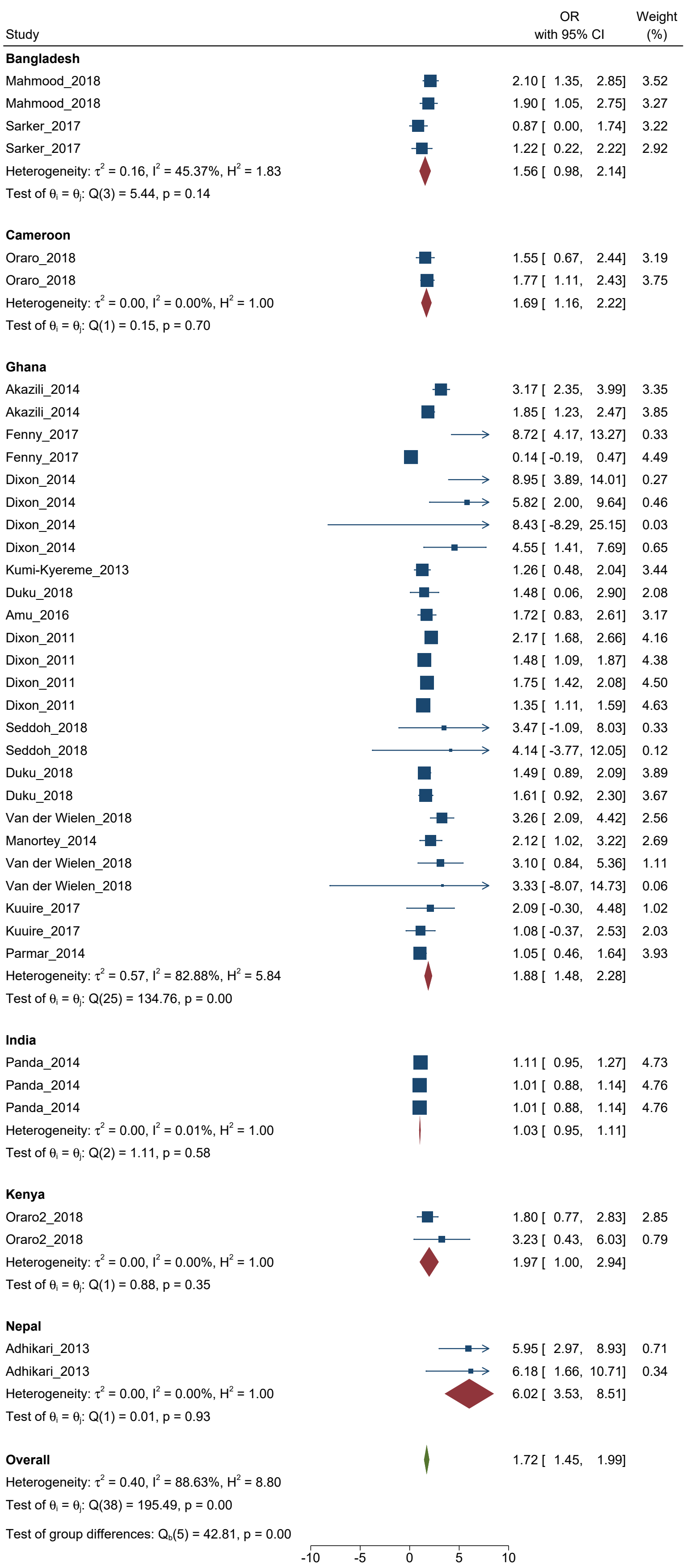

Supplement: Supplementary file 4 — Additional file 4. Figure S2. Forest plot showing countries with multiple estimates. [file 12939_2021_1608_MOESM4_ESM.pdf]

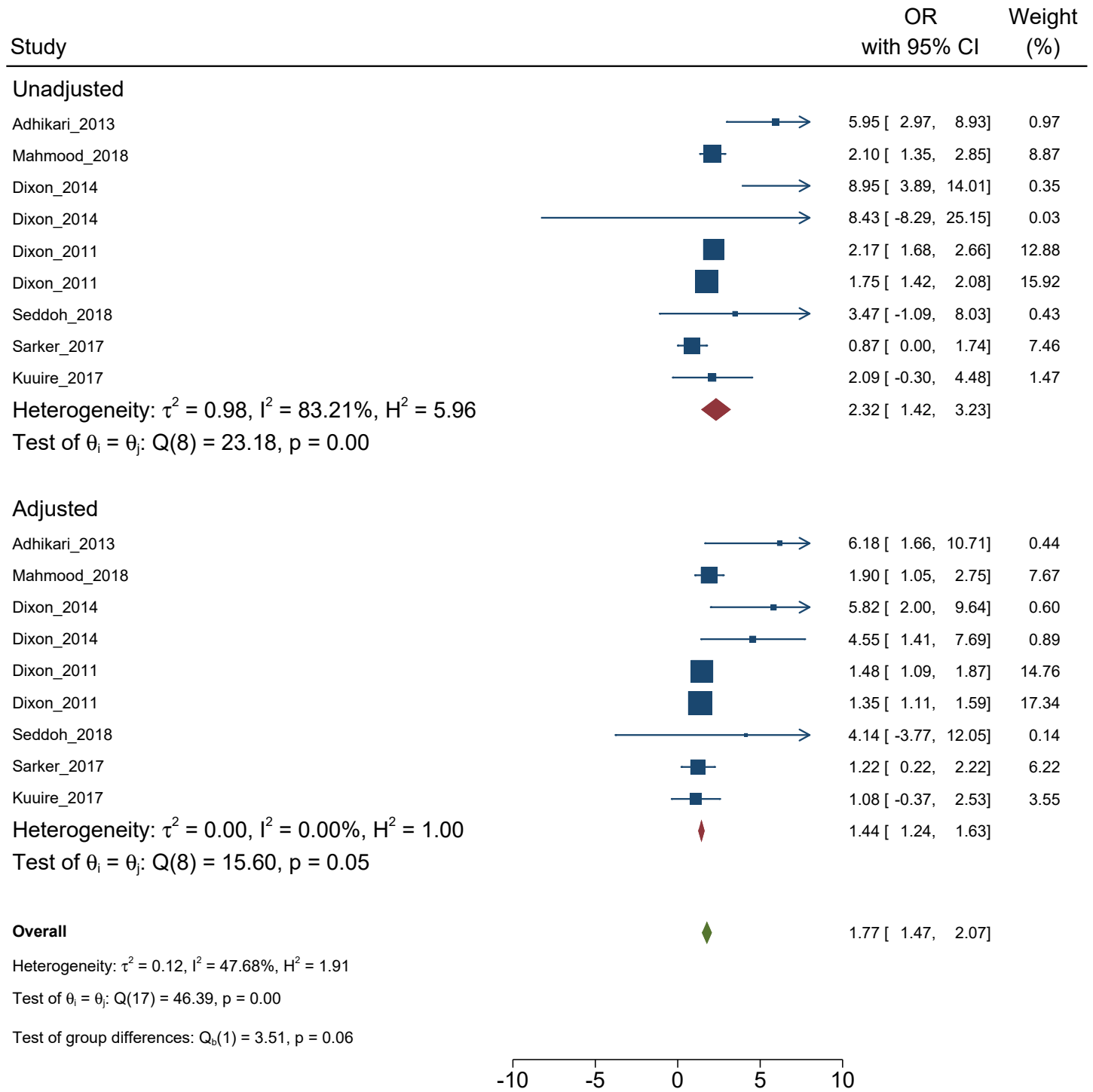

Random-effects REML model

Supplement: Supplementary file 7 — Additional file 7. Figure S5. Forest plot showing subgroup analysis of adjusted and crude odds ratio. [file 12939_2021_1608_MOESM7_ESM.pdf]
