# Supplementary material for: Equity in health insurance schemes enrollment in low and middle-income countries: A systematic review and meta-analysis
Source: Int J Equity Health. 2022 Feb 12;21:21. doi: 10.1186/s12939-021-01608-x (PMC8841076; doi:10.1186/s12939-021-01608-x)
Supplement: Supplementary file 5 — Additional file 5. Figure S3. Absolute percentage enrollment gap at the population level between the least and most educated groups. [file 12939_2021_1608_MOESM5_ESM.docx]

**Figure S3. Absolute percentage enrollment gap at the population level between the least and most educated groups**

Eighteen studies from nine countries reported the absolute health insurance differences at the population level between the lowest and highest education groups. However, we removed one low quality study and it remained with 17 studies. Fifteen of these studies reported a lower percentage of enrollment in health insurance schemes for the least educated groups than the most educated groups. This enrollment gaps between the least and most educated groups ranged from -6.9% to -40.6%. The two studies, which reported a higher percentage of enrollment in health insurance schemes for the least educated groups had a higher percentage gap of enrollment of 15.2 % whilst the lowest percentage gap was 3.1% [1, 2].

**References**

1. Govender V, Chersich MF, Harris B, Alaba O, Ataguba JE, Nxumalo N, Goudge J: **Moving towards universal coverage in South Africa? Lessons from a voluntary government insurance scheme**. *Global Health Action* 2013, **6**(1):109-119.

2. Boateng D, Awunyor-Vitor D: **Health insurance in Ghana: evaluation of policy holders' perceptions and factors influencing policy renewal in the Volta region**. *International journal for equity in health* 2013, **12**(50):1-10.
