# Supplementary material for: Equity in health insurance schemes enrollment in low and middle-income countries: A systematic review and meta-analysis
Source: Int J Equity Health. 2022 Feb 12;21:21. doi: 10.1186/s12939-021-01608-x (PMC8841076; doi:10.1186/s12939-021-01608-x)
Supplement: Supplementary file 8 — Additional file 8. File S6. Figures showing estimates regardless of quality rating. [file 12939_2021_1608_MOESM8_ESM.docx]

**Enrolment rate into health insurance schemes among vulnerable groups**

Notes: Enrollment rates correspond to reported enrollment rates in the lowest wealth quintile with the exception of Laos and South Africa, where data was only available for the lowest education (no formal education) group

**Absolute percentage enrollment gap at the population level between the least and most wealth groups**

**Absolute percentage enrollment gap at the population level between the least and most educated groups**
